# Deterministic and probabilistic radiological risks associated with gold mining activities in some villages along Jibia Niger–Nigeria border

**DOI:** 10.1007/s10653-025-02761-w

**Published:** 2025-09-26

**Authors:** Suleiman Bello, Muyiwa Michael Orosun

**Affiliations:** 1 Department of Physics, Umaru Musa Yar’adua University Katsina, Katsina, Nigeria; 2 Institute of Environmental Radioactivity, Fukushima University, Fukushima, Japan; 3 Physics Department, University of Ilorin, Ilorin, Nigeria

**Keywords:** Mining, RESRAD, Monte Carlo simulation, Dan Issa, Cancer risk

## Abstract

Deterministic and probabilistic methods were employed to assess the radiological risks associated with gold mining activities to both workers and the public in selected villages along the Jibia Niger–Nigeria border. In this study, a high-purity germanium detector was used to measure the specific activities of naturally occurring radioactive materials (NORMs). The Oracle Crystal Ball Monte Carlo simulation estimated the annual effective dose (AED) at 90 ± 50 µSv/yr, while the RESRAD (deterministic) simulation yielded an estimate of 240 µSv/yr. Similarly, the excess lifetime cancer risk (ELCR) was calculated to be 4.5E-4 ± 2E-4 through the Oracle crystal ball Monte Carlo simulation, and 7E-4 through RESRAD. While the effective dose values remain within the dose limits recommended by the ICRP, the estimated excess lifetime cancer risks suggest the need for implementing appropriate risk reduction strategies. It is recommended that radiation safety standards at mining sites be strengthened, public awareness be increased, and periodic environmental monitoring be conducted to mitigate long-term health risks.

## Introduction

Mining is the extraction of mineral deposits from the earth’s surface or subsurface, typically in regions with commercially viable reserves (Bello et al., 2022). Artisanal gold mining is an informal activity that employs millions worldwide, using basic tools to extract gold. While it supports livelihoods, it also causes significant environmental impacts (Machac, 2019). According to the World Bank (2023), nearly 45 million people, including miners’ families and supply-chain workers, depend on artisanal mining. In Niger Republic, this sector holds potential for socioeconomic development and economic diversification (World Bank, 2023).

Despite these benefits, artisanal mining is largely performed by unskilled workers (Nour et al., 2005), resulting in frequent health and safety risks. For example, a mine collapse in the study area in November 2021 killed at least 18 people (Reuters, 2021). Beyond such accidents, mining operations disturb naturally occurring radioactive materials (NORMs)—including ^22^⁶Ra (^23^⁸U), ^232^Th, and ^4^⁰K—which may create chemical and radiological hazards for miners and surrounding communities (UNSCEAR, 1993; Muhammad et al., 2024; Chinyere, 2019).

Residues from mining can elevate environmental radiation above background levels, exposing humans via inhalation, ingestion, or external pathways (UNSCEAR, 2009; Ziajahromi et al., 2014, 2021). Numerous global and African studies have assessed mining-related radiation (Gondi et al., 2022; Omotehinse & Ako, 2019; Usikalu et al., 2022; Sayyed et al., 2024; Wais et al., 2023, 2025a, 2025b), but radiological impacts remain site-specific due to geological and operational differences (Bello et al., 2022).

Quantifying radionuclide behaviour in soil, water, and food chains is crucial for estimating total radiation dose and excess lifetime cancer risk (ELCR) (Aliyu et al., 2015; Beauvais et al., 2009; Furo et al., 2024). The Residual Radioactivity (RESRAD) code—validated globally for environmental risk assessment—supports such evaluations (Yu et al., 2001; Wood, 2002).

*This study assessed activity concentrations of* ^*226*^ *Ra,* ^*238*^ *U,* ^*232*^ *Th, and* ^*40*^ *K and associated effective doses arising from gold mining activities in villages along the Jibia Niger-Nigeria border. Deterministic and probabilistic methods were used to estimate radiological risks to members of the public.*

## Materials and methods

### Sample collection and preparation

**Fig. 1:**
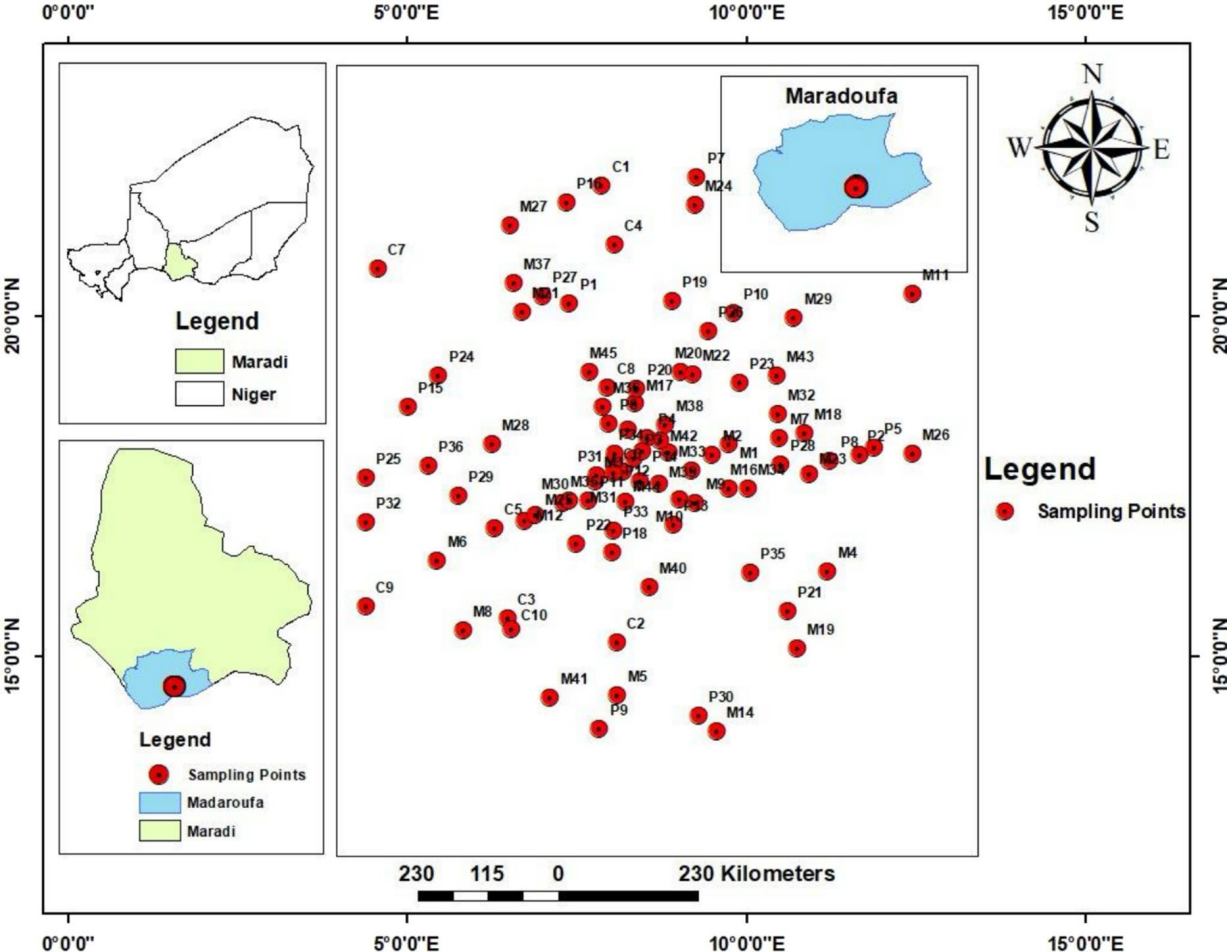
Map of the study area indicating sampling points

Artisanal gold mining and processing in the study area are concentrated in villages around Dan Issa, Niger Republic (Fig. 1). Soil excavation occurs primarily near Kwandago village, close to the Niger–Nigeria border in Jibia. Excavated soil is transported to Dan Issa, Madarounfa Local Government, for processing along the Maradi road. Sampling locations were selected for accessibility and proximity to mining sites, using a stratified random sampling strategy (IAEA, 2004; USEPA, 1989). Soil was chosen as the analytical matrix because it is a key reservoir and indicator of contamination.

A total of 91 representative soil samples were collected: 45 from active mining pits, 36 from processing zones (including tailings), and 10 from control sites located several kilometers upwind to minimize anthropogenic influence while maintaining comparable geology. This design captured radiological variability between operational zones and natural background, emphasizing areas of highest potential exposure.

The data showed positive skewness due to localized radioactivity hotspots, typical of artisanal mining with non-uniform waste handling. This highlights the need for future spatially stratified sampling. Radon-222 (^222^Rn) and its progeny were not measured because of logistical constraints and limited equipment access, though future studies will include radon monitoring in soil and indoor air to provide a fuller exposure profile.

Samples were sealed in polyethylene bags, labeled, and prepared by removing foreign material, oven drying at ~ 110 °C for 12–18 h, pulverizing, and sieving through a 2 mm mesh. Homogenized samples (25 g) were placed in plastic containers (7.2 cm × 6 cm), hermetically sealed with PVC tape to prevent ^222^Rn/^22^⁰Rn escape, and stored for at least 24 days to achieve secular equilibrium. Gamma spectrometry calibration and quality control employed IAEA reference materials (RGK-1, IAEA-448, RGTh-1), consistent with standard radiometric protocols.

### ***Experimental determination of the activities of ***^***238***^***U, ***^***232***^***Th and ***^***40***^*** K***

The prepared soil samples were analyzed using a high-purity germanium (HPGe) detector, shielded with 0.16 cm copper, 0.1 cm tin, and a 10 cm lead enclosure to reduce background radiation interference (IAEA, 2004). The detector was calibrated prior to analysis using a multi-nuclide source containing ^13^⁷Cs, ^60^Co, ^5^⁷Co, and ^22^Na to establish accurate energy and efficiency settings (Gilmore, 2008). Spectral data were processed using CANBERRA Genie 2000 software, with background radiation subtracted from each sample spectrum to enhance measurement precision. Radionuclide activities were quantified using characteristic gamma-ray peaks: ^4^⁰K at 1461 keV (10.8% emission probability), ^22^⁶Ra via ^214^Bi at 1764 keV (15.3%), and ^232^Th via ^2^⁰⁸Tl at 2615 keV (9.7%) for samples, standards, and background measurements (IAEA, 2004). To monitor environmental radiation variability, background counts were performed every three days using an empty container matching the sample container geometry, serving as a reference for background correction. Peak energy efficiency was calculated per Eq. 1, incorporating the detector’s detection limit (DL) and minimum detectable activity (MDA), determined using Eqs. 2 and 3 (IAEA, 2004), based on counting time and background rates. These procedures ensured robust and reliable quantification of radionuclide concentrations, adhering to best practices for environmental radiological studies.

1 $$\varepsilon = \frac{N}{\gamma AB}$$

2 $$DL=2.71+4.66\sqrt{BN}$$

3 $$MDA=\frac{DL}{\gamma \varepsilon BM}$$

where N (count s⁻^1^) represents the net count rate, γ (s) denotes the counting time, A (Bq) is the sample activity, B indicates the branching ratio, BN (count s⁻^1^) signifies the background count rate, and M (kg) is the mass of the sample or standard (IAEA, 2004). The total photopeak efficiencies for the gamma energies of 1764 keV (^214^Bi for ^22^⁶Ra), 1461 keV (^4^⁰K), and 2615 keV (^2^⁰⁸Tl for ^232^Th) were 0.02, 0.06, and 0.005, respectively. The detection limits (DL) for these energies were 67 ± 0.5, 86 ± 4, and 112 ± 2 counts, respectively, while the minimum detectable activities (MDA) were 5.3 ± 0.1, 6.2 ± 0.1, and 22.1 ± 0.2 Bq kg⁻^1^ for ^22^⁶Ra, ^4^⁰K, and ^232^Th, respectively to ensure measurement sensitivity.

The activity concentration of radionuclides in the soil samples was determined by comparative analysis using a certified reference material (IAEA Soil-375) characterized by known radionuclide concentrations. The reference material was measured under identical conditions as the unknown samples and the blank, using the same geometry and duration (12 h). Net count rates (counts per second) corresponding to the characteristic gamma photo peaks of the target radionuclides were extracted from both the reference and the samples. These values were substituted into Eq. (4) to compute the activity concentrations of the radionuclides in the unknown samples:

4 $${C}_{s}=\frac{{M}_{ref}\times {N}_{s}}{{M}_{s}\times {N}_{ref}}\times {C}_{ref}$$

where, C_s_ = sample’s concentration (Bq/kg), M_ref_ = mass of the reference (Soil-375) material (kg), N_ref_ = Net count of reference material, M_s_ = mass of the soil sample (kg), C_ref_ = reference material’s concentrations (Bq/kg), and N_s_ = net count of the soil sample.

### Statistical analysis

The results obtained for activity concentrations were analyzed statistically using SPSS (version 23). Descriptive statistics and box plots were employed to summarize the findings, providing insights into the data distribution and central tendencies. Furthermore, analysis of variance (ANOVA) and correlation analysis were conducted to explore potential relationships and differences between samples collected from the target and control areas.

According to the United States Geological Survey, environmental datasets are often characterized by censored values, non-normal distributions, outliers, and skewness. In line with this, the dataset in this study was subjected to normality testing, and in some cases, median values and standard errors were computed to better represent the population (Ewuzie et al., 2021; Helsel et al., 2020). The Shapiro–Wilk test was used to assess normality, revealing deviations from normal distribution in certain variables.

The box plot was interpreted as follows: the whiskers represent the minimum and maximum values of the dataset, excluding outliers. The central line within the box indicates the median (Q2), where 50% of the data lie below and 50% above this value. The lower and upper edges of the box correspond to the first quartile (Q1) and third quartile (Q3), which represent the 25th and 75th percentiles, respectively—meaning 25% of the data fall below Q1 and 75% below Q3 (Kanmi et al., 2025).

Skewness analysis was also performed to assess the symmetry of the data distribution. A skewness value of zero indicates perfect symmetry. Positive skewness signifies a right-skewed distribution, characterized by a longer tail on the right, suggesting the presence of higher values or outliers that shift the distribution toward larger values. This implies a concentration of data points at the lower end, with a few extreme values extending the right tail (Namq et al., 2025).

### Deterministic radiological risks assessment using RESRAD

**Fig. 2:**
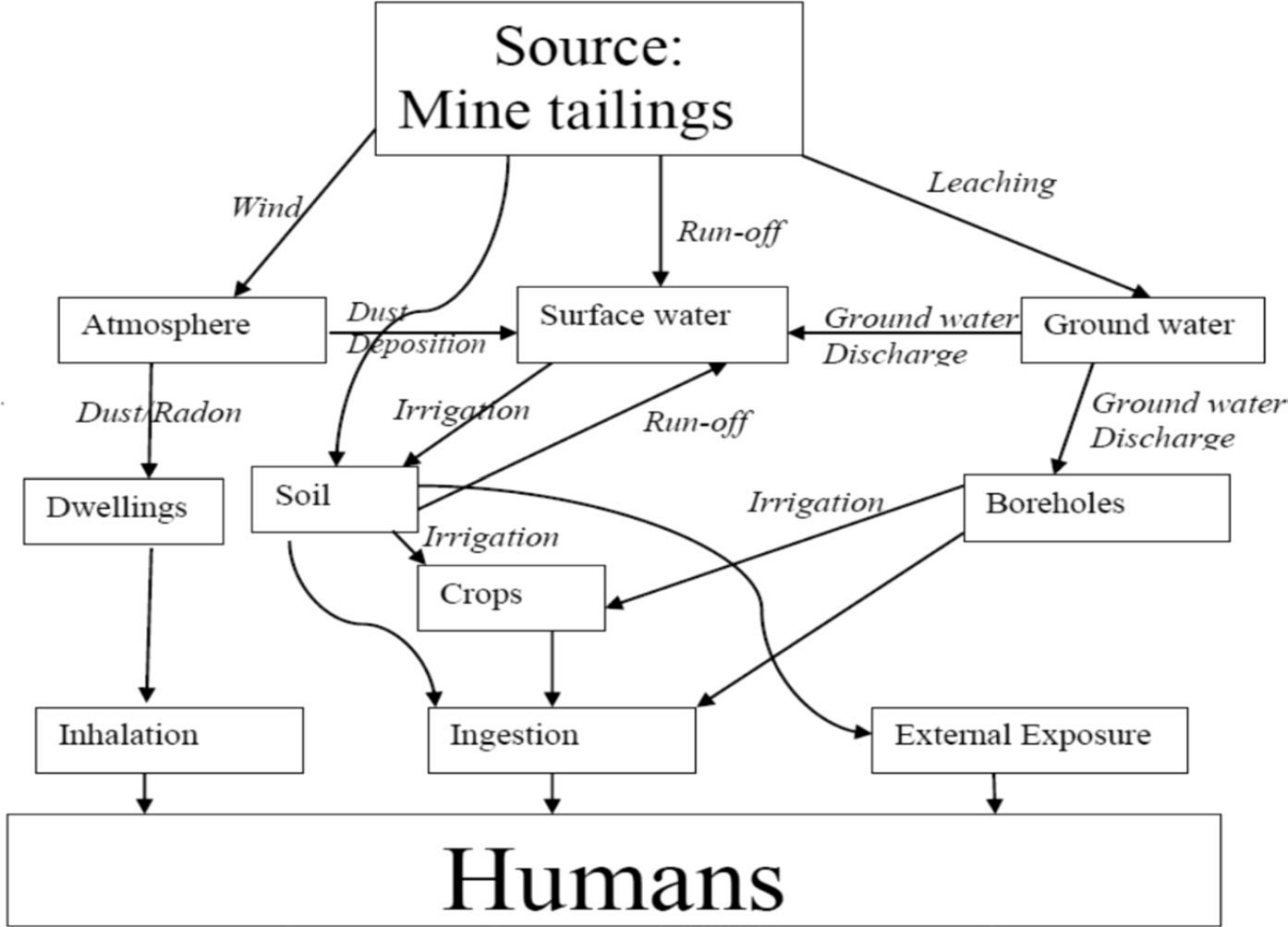
Typical existing residential exposure scenario as adopted from Mathuthu et al., (2016)

Deterministic radiological dose and risk calculations were performed using RESRAD ONSITE (version 7.2, Argonne National Laboratory, 9700 South Cass Avenue, EVS/900, Argonne, IL 60439) on a 2.4 GHz HP personal computer (11th Gen Intel® Core™ i5-1135G7). A typical residential exposure scenario, as depicted in Fig. 2, was adopted for the computations, as it accurately represents the exposure conditions of the study area and provides a more realistic modeling of dose and risk.

Radiological dose assessments incorporated both water-independent pathways—based on soil radionuclide concentrations—and water-dependent pathways—relying on radionuclide levels in groundwater—following established simulation methodologies (Yu et al., 2001). These exposure scenarios offered a consistent and conservative framework for evaluating multiple routes of exposure to Naturally Occurring Radioactive Materials (NORMs) resulting from artisanal gold mining activities.

To enhance model accuracy, site-specific parameters such as soil activity concentrations, hydrological and geological characteristics, and dietary and lifestyle habits of the local population were incorporated where available (Hagemann, 2013). Table 1 outlines the modifications made to default parameters to better reflect conditions in the study area and comparable regions. Notably, due to the lack of electricity in the area, storage times prior to consumption were adjusted to reflect local practices: seven days for leafy vegetables and water, three days for milk, meat, and fish, and 90 days for livestock fodder (Bello et al., 2022).

**Table 1:** Modifications on some default RESRAD ONSITE parameters

S/N	Parameter	Value	Description and/or reference
1	Soil activity concentration of ^238^U (^226^Ra), ^232^Th, ^40^ K (Bq/kg)	26.11 ^238^U; 17.66 ^32^Th; 345.30 ^40^ K	Median values of the determined activity concentrations from the site
2	Cover depth (m)	0	There was no cover depth for the radionuclides
3	Density of contaminated zone (g/m^3^)	1.44	(Yani et al., 2023)
4	Precipitation rate (m/year)	1	Relatively humid (NNR, 2013)
5	Runoff coefficient	0.65	For moderately steep residential area (Yu et al., 1993)
6	Inhalation rate (m^3^/year)	8400	Resident farmer scenario (Yu et al*.,* 2001)
7	Hydraulic conductivity (m/year)	1090	For sandy loam soil (Yu et al., 1993)
8	Average wind speed (m/s)	4.1	Maximum for Nigeria (Olalekan et al., 2020)
9	Soil specific exponential b parameter	4.9	For sandy loam soil (Yu et al., 1993)
10	Water drinking rate (L/year)	730.5	ICRP standard man concept (Bello et al*.,*2020)
11	Well pump intake depth (m)	10	Based on data from south Africa (Njinga & Tshivhase, 2018)
12	Outdoor/indoor occupancy factor	0.5/0.3	Estimated based on the social habits of a typical resident farmer in the study area
13	Exposure duration	30 years	Resident farmer scenario (Yu et al., 2001)
14	Soil ingestion rate (g/year)	37	Based on data from south Africa (Mathuthu et al., 2016)

For radiological protection purposes, only parent and daughter radionuclides with half-lives exceeding 30 days were considered. Dose calculations were carried out at time intervals of 0, 1, 30, 50, 70, 100, and 1000 years to assess long-term exposure impacts (Limen & Makondelele 2018). Dose conversion factors (DCFs), transfer factors, and bioaccumulation coefficients were sourced from the DCFPAK 3.02 morbidity/mortality library, consistent with ICRP Publication 107 (ICRP, 2007). An exposure duration of 30 years was assumed, with a regulatory dose limit of 0.25 mSv/year (Yu et al., 2001).

To standardize assessments, the ICRP reference man (20–30 years of age, 70 kg body weight, 170 cm height, Caucasian, Western European/North American lifestyle, residing in a 10–20 °C climate) was adopted as the receptor model (ICRP, 1975), avoiding the complexity of age- and gender-specific calculations. Dose coefficients for ^222^Rn and ^220^Rn inhalation were obtained from the BEIR IV report and ICRP Publications 32 and 47 (Yu et al., 2001).

Active exposure pathways included external gamma radiation, inhalation, and ingestion of plants, meat, milk, aquatic foods, drinking water, soil, and radon, ensuring a comprehensive estimate of total radiation dose. The occupancy factor was adjusted to reflect the rural nature of the study area, where outdoor activities predominate. The default occupancy values, based on urban and temperate region lifestyles with predominantly indoor time budgets, were deemed unsuitable for this context (Arogunjo et al., 2004). These adjustments ensured a robust and context-sensitive radiological risk assessment for the artisanal gold mining environment.

### Probabilistic radiological risks assessment using RESRAD

In addition to the deterministic radiological risk assessment, a probabilistic risk assessment was conducted using the same RESRAD specifications described earlier. For the probabilistic analysis, plausible probability distributions were assigned to each selected uncertain parameter used in the dose calculations, as presented in Table 2. To estimate dose distribution functions, a stratified Monte Carlo Latin Hypercube Sampling (LHS) technique was employed, providing an efficient approach for analyzing multiple parameters (Kamboj et al., 2000). This method ensures comprehensive sampling across the full range of each parameter, producing dose estimates expressed as quantile values derived from the resulting analysis.

**Table 2:** Assigned probability distribution for uncertain parameters

S/N	Parameters	Assigned distribution
1	Density of contaminated zone. Density of cover material, density of saturated zone,	Normal
2	Distribution coefficient, saturated zone hydraulic conductivity, transfer factors for plants, wind speed	Log normal
3	Evapotranspiration coefficient	Uniform
4	Fruits, vegetables and grains consumption, well pump intake depth, inhalation rate, occupancy factor	Triangular
5	Cover depth, well pumping rate	No recommendation

Regression analysis was also performed to identify the most sensitive parameters influencing dose outcomes (Kamboj et al., 2000). The RESRAD model was executed for 10,000 iterations, each utilizing randomized values for the uncertain input parameters, with the results stored for further analysis. Outputs—including dose and cancer risk estimates—were generated by randomly selecting from the stored model results and presented as probability density functions (PDFs) and cumulative distribution functions (CDFs). The CDFs provided a quantitative interpretation of percentile values within the dose and risk distributions.

Additionally, a single-parameter sensitivity analysis was conducted using RESRAD to evaluate the influence of key parameters—specifically, contamination zone thickness, cover depth, occupancy factor, inhalation rate, soil ingestion rate, water consumption rate, and cancer risk—on the resulting radiological exposure (Mathuthu et al., 2016). This analysis offered valuable insights into the parameters with the greatest impact on risk variability.

### Probabilistic radiological risks assessment using oracle crystal ball

Uncertainties in activity concentrations and exposure input parameters can result in either overestimation or underestimation of radiological dose and risk (Karami et al., 2019; Omeje et al., 2022; Orosun et al., 2022; Shahrbabki et al., 2018). To address this, Monte Carlo simulations were carried out using Oracle Crystal Ball (version 11.1.4716.0, Oracle, Denver, CO, USA). Probability distributions for each input parameter were derived from established literature to capture the inherent uncertainty and variability (Karami et al., 2019; Ramesh et al., 2021; Seyedeh et al., 2023).

Random sampling from these distributions, as detailed in Table 3, was performed over 10,000 iterations to ensure result stability and convergence, with a 95% confidence level applied to approximate the probabilistic risk assessment (Shi et al., 2022). The resulting exposure distributions were analyzed across various quartiles to evaluate probabilistic radiological risk (Yang et al., 2019).

**Table 3:** Values used in the MC Simulation in Oracle crystal ball

Parameter	Distribution assumed	Average	Standard deviation	Reference
^238^U (^226^Ra), ^232^Th and ^40^ K mean/median activity concentrations (Bq/kg)	Log Normal	26.11; 17.66 and 345.30	^238^U (8.92); ^232^Th (8.100) and ^40^ K (331.93)	Present study
Total AED (µSv/yr)	Log Normal	104.73	62.57	Present study
Outdoor occupancy factor	Triangular	0.2 (min)	0.5 (max)	
Duration of life (years)	Normal	70	10	Default
Risk factor	Uniform	0.05		Present study

Sensitivity analysis was also conducted to identify the most influential input parameters and quantify their respective contributions to uncertainty in the excess lifetime cancer risk (ELCR). Positive correlation coefficients indicated parameters that increased risk, while negative coefficients suggested an inverse relationship with ELCR.

To validate the findings, ELCR values obtained from the Monte Carlo simulations were compared with those derived from deterministic calculations. Multiple independent simulation runs—each with 10,000 iterations—were conducted to confirm the robustness and reproducibility of the ELCR estimates, enhancing the reliability of the overall risk assessment.

## Results and discussion

### ***Activity concentration of ***^***238***^***U (***^***226***^***Ra), ***^***232***^***Th and ***^***40***^*** K***

**Fig. 3:**
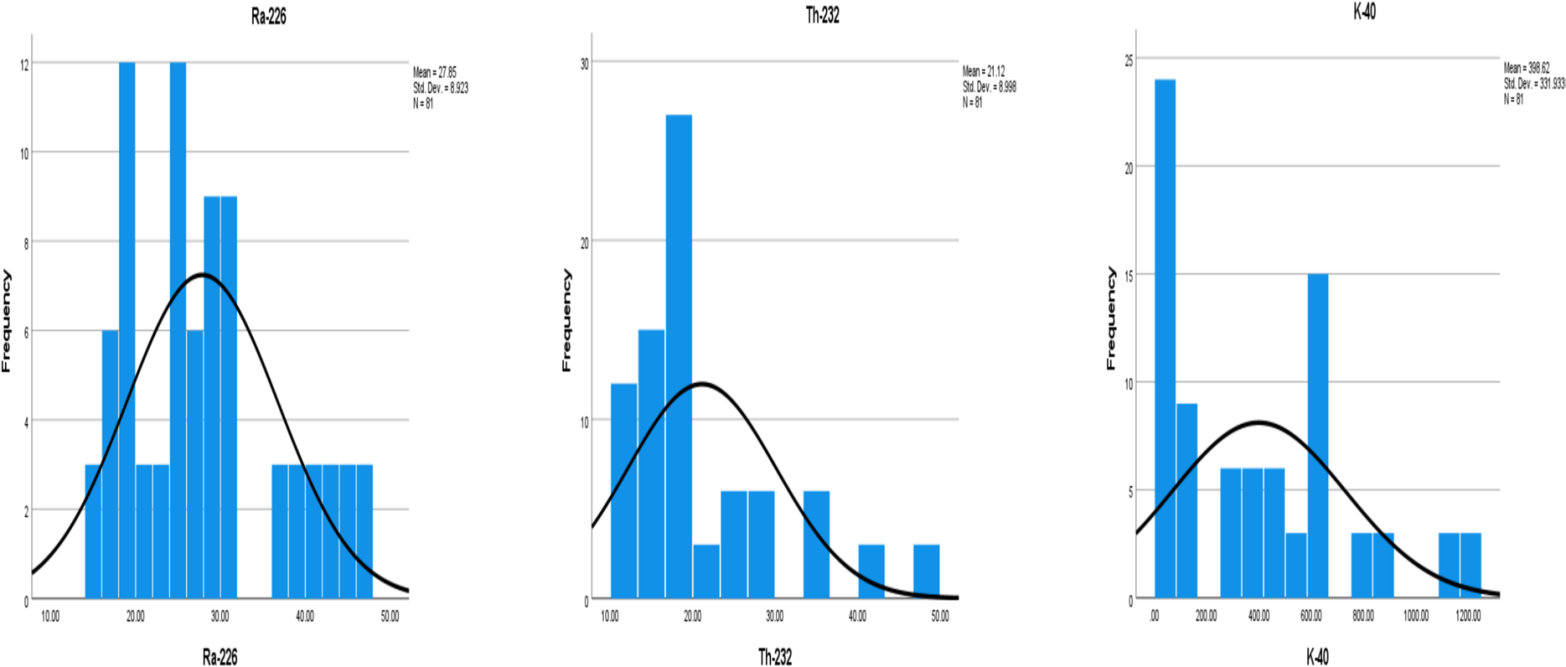
Activity concentrations (Bq/kg) of ^238^U (^226^Ra), ^232^Th and ^40^ K

Table 4 presents the descriptive statistics for the average concentrations of Naturally Occurring Radioactive Materials (NORMs) in soil samples collected from the gold mining and processing zones in Dan-Issa, Maradi, Niger Republic. To assess the distribution characteristics of the measured activity concentrations, histograms were generated with superimposed normal curves (Fig. 3a–c), allowing for a visual evaluation of normality. To comprehensively describe the statistical properties of the activity concentrations, the mean, median, standard deviation, skewness, and kurtosis values were calculated and are also presented in Table 4.

**Table 4:** Descriptive statistics of the specific activity of NORMs (Bq/kg)

Statistics				
		Ra-226	Th-232	K-40
N	Valid	81	81	81
Mean		27.8544	21.1204	398.6207
Std. Error of Mean		0.99139	0.99974	36.88145
Median		26.1100	17.6600	345.3000
Std. Deviation		8.92252	8.99770	331.93305
Skewness		0.688	1.415	0.777
Std. Error of Skewness		0.267	0.267	0.267
Kurtosis		− 0.451	1.314	− 0.212
Std. Error of Kurtosis		0.529	0.529	0.529

The activity concentration of the NORMs (^238^U (^226^Ra), ^232^Th and ^40^ K) for all the samples from the mining and processing zones were determined to be 27.85 ± 8.92, 21.12 ± 8.10 and 398.62 ± 331.93 Bq/kg respectively. In all the radionuclides considered; the activity concentration in the target areas (mining and processing zones) were greater than the values obtained from a carefully selected control area. The control area had ^238^U (^226^Ra), ^232^Th and ^40^ K activity concentrations of; 19.33 ± 55.49, 14.15 ± 13.01 and 46.53 ± 55.49 Bq/kg respectively. The individual activity concentrations evidently had a wide variation from the central value, as is usually the case in environmental samples.

The average worldwide activity concentration of ^238^U (^226^Ra), ^232^Th and ^40^ K is 35 Bq/kg, 30 Bq/kg and 400 Bq/kg respectively. The activity concentration of these radionuclides in the analysed samples were less than the worldwide activity concentrations in most of the samples (> 50%). However; 22% of the samples had an activity concentration of ^238^U (^226^Ra) greater than the global average, 15% of the samples had an activity concentration of ^232^Th greater than the global average, and 44% of the samples had an activity concentration of ^40^ K activity concentration greater than the global average. Most of the samples exhibited NORMs activity concentrations below the worldwide average, suggesting that gold mining activities did not significantly elevate the natural radioactivity levels in the soil. Notably, the tailing site recorded higher values compared to the mine site. Among the radionuclides, ^40^ K exhibited the highest activity concentration, potentially due to its relatively abundant presence as a macro element in soil. Importantly, this elevated concentration does not pose a health concern as homeostasis maintains it at a constant level in living organisms. It should be noted that a major limitation of this study is the skewed sampling distribution, therefore, future investigations should aim to increase the number of samples to ensure a normally distributed dataset, thereby enhancing representativeness. Moreover, the comparison of soil samples collected from the mining area, processing area, and the control area using analysis of variance (ANOVA) revealed no statistically significant difference in the activity concentrations of the NORMs among the studied sites (P < 0.05). Correlation analysis demonstrated a significant positive correlation between ^238^U (^226^Ra) and ^232^Th in the mining area (r = 0.93), tailing area (r = 0.85), and control area (r = 0.96), indicating that the activity concentration of ^238^U (^226^Ra) increases or decreases with that of ^232^Th in all considered areas. Furthermore, in the tailing area, a significant correlation between ^238^U (^226^Ra) and ^40^ K (r = 0.85) was observed, suggesting that the activity concentration of ^232^Th increases with an increase in the activity concentration of ^238^U (^226^Ra). The results of the one-sample analysis of variance (ANOVA) test indicate that the P-value for uranium, thorium, and potassium is 0.000. This finding confirms that the measured activity concentrations of the NORMs differ significantly, further substantiating the unequal median values observed, which may be attributed to their relative abundance.

### Annual effective dose

For a more comprehensive radiological risk assessment, deterministic assessment was used to estimate the dose due to its additional advantage of including more pathways and routes in the estimation of doses (external, inhalation, radon, plant, meat, milk, drinking water and fish). The results from RESRAD Onsite indicated that the dose from all the considered nuclides and component pathways for the considered calculation times (0–1000) years, is all less than the basic safety limit of 0.25 mSv/yr (Yu et al., 2001) and the 1 mSv/yr annual effective dose as set by ICRP and adopted in Nigeria**.** However, the results indicated that the water independent pathways will present a dose greater than 0.25 mSv/yr between 2 and 70 years.

**Fig. 4:**
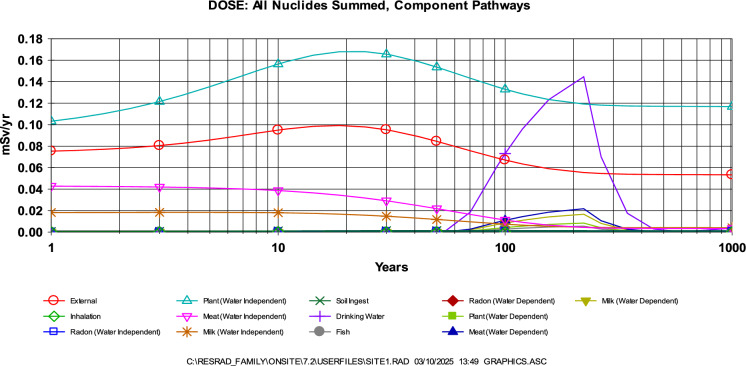
Total dose from all the nuclides and pathways

**Fig. 5:**
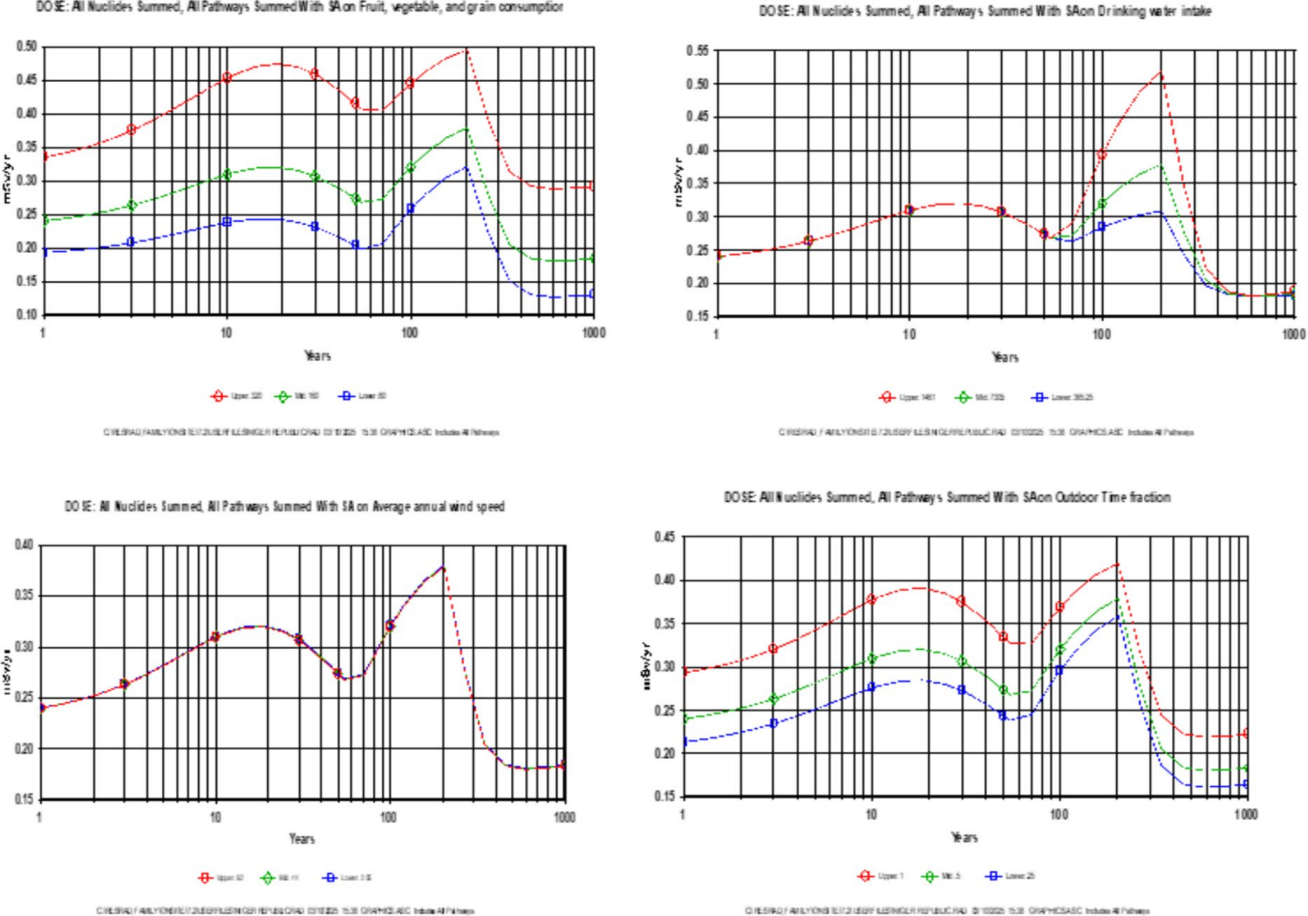
Sensitivity analysis output from RESRAD

Probabilistic assessment on the other hand, indicated that the total dose received was however greater than the basic radiation dose limit of 0.25 mSv/yr (Yu et al., 2001) for 1, 3, 10, 30, 50 and 100 years. The maximum total dose occurred at 221.9 ± 0.4 years which is 3.826E-1 mSv/yr. This indicates that to ensure protection of future generation, there is need to clean up gold mine pits to at least bring down the dose to the basic radiation dose limit by covering it with clean soil. The results of the deterministic simulation were further corroborated by that of the probabilistic simulation as indicated in the cumulative probability summary for total dose over pathways presented in Fig. 4. Though, one of the limitations of this result is that limited site specific and receptor specific information was used during the simulation. Some parameters that were used—which have proven to be a potential cause of possible overestimation/underestimation of this values were subjected to sensitivity analysis. Figure 5 presented the impact of some of these variables to the estimated dose (fruits, vegetable and grain consumption, drinking water intake, average annual wind speed and outdoor time fraction). The obtained AED for t = 1 year corroborates the findings of Bello et al., (2019); Esiole et al., (2019); Ayua et al., (2017); Gomina et al., (2020) and was far less than Ademole et al., (2014); Joseph et al., (2022); Kileo et al., (2025) and Saidou et al., (2019).

### Excess lifetime cancer risk

The International Commission on Radiological Protection (ICRP) maintains that no level of radiation exposure is entirely without risk, as even minimal doses can induce stochastic effects such as cancer. Therefore, beyond adhering to dose limits, the ALARA principle (“As Low As Reasonably Achievable”) remains central to radiological protection. According to the United States Environmental Protection Agency (USEPA), acceptable cancer risk levels range from 1E-6 to 1E-4.

**Fig. 6:**
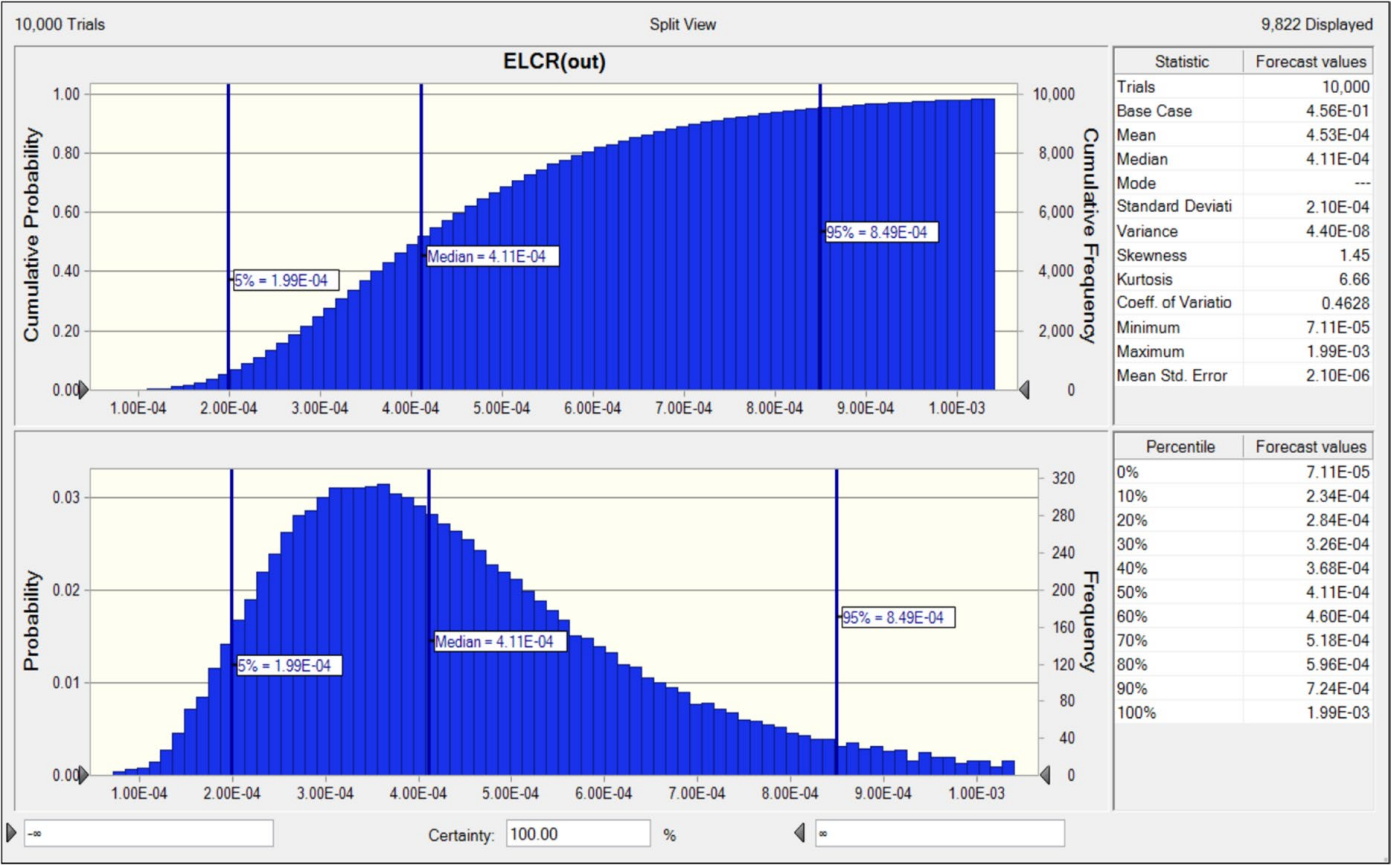
Cumulative probability plot for ELCR as determined through MC simulation

In this study, the total excess lifetime cancer risk (ELCR) from ingestion and inhalation of ^238^U (^226^Ra), ^232^Th and ^40^ K had a median ELCR value of 4.11E-4 as estimated through the Oracle-based Monte Carlo simulation (Fig. 6). This value exceeded the upper bound of the USEPA acceptable range.

**Fig. 7:**
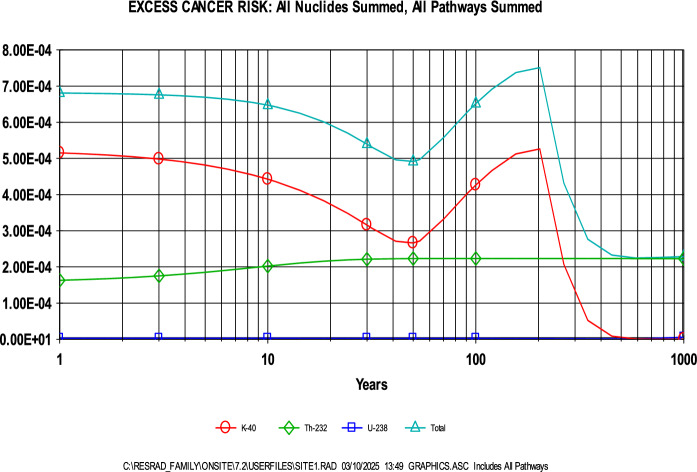
Contribution of radionuclides to the ELCR as determined through RESRAD

**Fig. 8:**
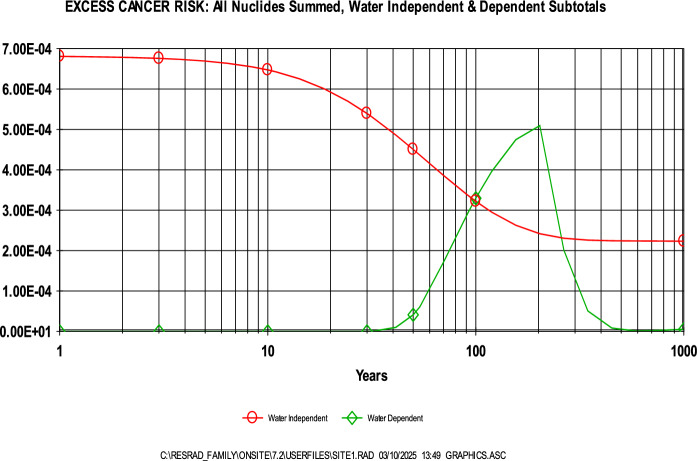
Contribution of water dependent and independent pathways to ELCR

**Fig. 9:**
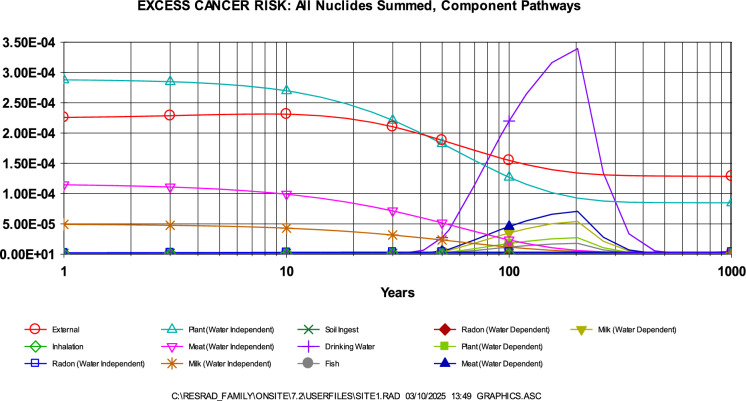
Contribution of different exposure component pathways to the ELCR

Further support was provided by RESRAD deterministic modeling, which estimated ELCR values ranging from 5E-4 to 7E-4 over a 0–100-year period. As with the annual effective dose, ^40^ K contributed most significantly to the risk, followed by ^232^Th (Fig. 7). The dominant exposure pathway during this period was found to be water-independent (Fig. 8), with pathway contributions over time illustrated in Fig. 9. These ELCR estimates are consistent with those reported by Kileo et al. (2025) and Bello et al. (2022), and are lower than the values found in Bello et al. (2019). However, it is important to recognize the potential uncertainty in these results due to the use of generalized or non-site-specific parameters. Notably, age-independent calculations, the application of a residential exposure scenario, and limited local data on sensitive input variables may result in either underestimation or overestimation of the actual cancer risk.

To strengthen future assessments and reduce uncertainty, site-specific values for key parameters—including soil-to-plant transfer factors, dietary intake rates, occupancy patterns, water consumption rates, and distribution coefficients—should be established. Additionally, age- and gender-specific risk modeling could improve the accuracy of ELCR projections, especially for vulnerable populations such as children and pregnant women.

## Conclusion

This study evaluated radiological risks from gold mining activities along the Jibia Niger–Nigeria border using deterministic and probabilistic methods. The activity concentrations of ^238^U (^226^Ra), ^232^Th and ^40^ K in mining and processing zone samples were 27.85 ± 8.92, 21.12 ± 8.10, and 398.62 ± 331.93 Bq/kg, respectively, all within UNSCEAR safety limits.

The estimated annual effective dose was 90 ± 50 µSv/yr (Monte Carlo) and 240 µSv/yr (RESRAD), remaining below the ICRP public dose limit of 1 mSv/yr, indicating negligible deterministic health effects. However, the excess lifetime cancer risk values, 4.5 × 10⁻^4^ ± 2 × 10⁻^4^ (Monte Carlo) and 7 × 10⁻^4^ (RESRAD), exceed the U.S. EPA benchmark of 1 × 10⁻^4^, implying a non-negligible probability of long-term stochastic effects, particularly cancer, among exposed populations.

## Recommendations

Although activity concentrations and annual effective doses from gold mining fall within acceptable limits, the elevated excess lifetime cancer risk (ELCR) calls for proactive mitigation. The following stakeholder-specific recommendations are proposed:

1. Gold Mining Company

- Conduct regular radiological monitoring and provide protective equipment for workers.
- Remediate high-risk areas (e.g., tailings, processing zones) using soil capping, waste control, or phytoremediation.
- Enforce occupational hygiene to reduce ingestion and inhalation of contaminants.

2. Nigeria and Niger Nuclear Regulatory Authorities

- Integrate radiological risk assessment into licensing and inspection protocols.
- Institutionalize routine monitoring of soil, water, and air in mining zones.
- Enforce zoning and land-use restrictions to limit residential development near contaminated sites.

3. Government Mining and Environmental Oversight Institutions

- Develop and enforce radiological protection guidelines tailored to artisanal mining.
- Establish health surveillance programs and conduct public awareness campaigns.
- Strengthen inter-agency collaboration to align mining activities with public and environmental safety goals.

## Data Availability

The datasets used and/or analyzed during the current study available from the corresponding author on reasonable request.

## Declarations

### Plant guideline statement

Experimental research and field studies on plants, including the collection of plant material, complied with relevant institutional, national, and international guidelines and legislation.

### Statement of novelty of the study

This study is among the first to integrate both deterministic and probabilistic approaches—including Monte Carlo simulations, and RESRAD modeling—to comprehensively assess radiological risks associated with artisanal gold mining. The multi-model comparison provides deeper insights into the variability and reliability of dose and cancer risk estimates in under-researched communities.

### Statement of industrial relevance

The findings offer critical implications for the mining industry, particularly in risk-prone artisanal mining regions. The results can inform policy makers, environmental health authorities, and mining operators on the need for improved radiation protection standards, site remediation practices, and public health surveillance. The approach used in this study may also serve as a model for similar risk assessments in comparable industrial settings.
